# Reward and aversion encoding in the lateral habenula for innate and learned behaviours

**DOI:** 10.1038/s41398-021-01774-0

**Published:** 2022-01-10

**Authors:** Sarah Mondoloni, Manuel Mameli, Mauro Congiu

**Affiliations:** 1 The Department of Fundamental Neuroscience, The University of Lausanne, 1005 Lausanne, Switzerland; 2 Inserm, UMR-S 839, 75005 Paris, France

**Keywords:** Learning and memory, Molecular neuroscience

## Abstract

Throughout life, individuals experience a vast array of positive and aversive events that trigger adaptive behavioural responses. These events are often unpredicted and engage actions that are likely anchored on innate behavioural programs expressed by each individual member of virtually all animal species. In a second step, environmental cues, that are initially neutral, acquire value through the association with external sensory stimuli, and become instrumental to predict upcoming positive or negative events. This process ultimately prompts learned goal-directed actions allowing the pursuit of rewarding experience or the avoidance of a danger. Both innate and learned behavioural programs are evolutionarily conserved and fundamental for survival. Among the brain structures participating in the encoding of positive/negative stimuli and contributing to innate and learned behaviours is the epithalamic lateral habenula (LHb). The LHb provides top-down control of monoaminergic systems, responds to unexpected appetitive/aversive stimuli as well as external cues that predict the upcoming rewards or punishments. Accordingly, the LHb controls a number of behaviours that are innate (originating from unpredicted stimuli), and learned (stemming from predictive cues). In this review, we will discuss the progresses that rodent’s experimental work made in identifying how LHb activity governs these vital processes, and we will provide a view on how these findings integrate within a complex circuit connectivity.

## Introduction

Unexpected experiences may lead to positive or negative outcomes, and virtually all animal species can innately and promptly execute specific behaviours after presentation of unpredicted stimuli—either stay and enjoy the positive outcome or alternatively escape the negative situation. Therefore, the brain can integrate external events and generate actions after experiencing situations until that moment unknown. When such events repeat over time instead, and external cues predict upcoming rewards or threats, individuals use learning processes to anticipate outcome occurrence and perform actions ahead of time. Similarly to the innate behavioural response, the expression of such anticipatory learned behaviours is fundamental for the survival of individuals. Hence, the brain is able to generate innate behaviours that are independent from previous experience, and actions that instead anchor to learning processes (learned behaviours).

**Fig. 1:**
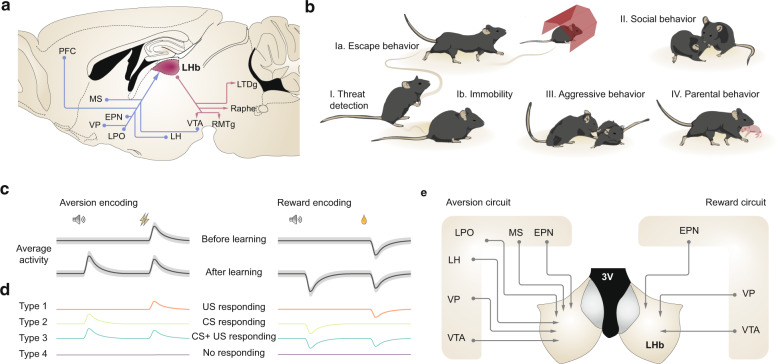
The LHb guides innate and learned behaviours by encoding rewarding and aversive stimuli through complex circuit connectivity and diversity in neuronal responses. **a** Schematic representing the principal LHb inputs (blue) and outputs (purple) contributing to innate and learned behaviours. Ventral tegmental area (VTA), lateral hypothalamus (LH), lateral preoptic area (LPO), ventral pallidum (VP), entopeduncular nucleus (EPN), medial septal nucleus (MS) and Prefrontal Cortex are projections to the LHb. LHb neurons send axons to VTA, Rostromedial Tegmental Nucleus (RMTg), Raphe and Laterodorsal Tegmental nucleus (LDTg). **b** Schematic representation of various innate behaviours that are controlled by LHb. Response to a threat (I.) and subsequent escape behaviour (Ia.) or immobility (Ib); social interactions (II.), aggressive interactions (III.) and parental behaviours (IV.). **c** Illustration of the average neuronal activity of LHb cells in response to a conditioned stimulus and unconditioned aversive and rewarding stimulus before and after associative learning. **d** Tentative model representing the neuronal and functional diversity present during the encoding of conditioned stimuli and unconditioned stimuli. Such a neuronal diversity is represented by the type I (orange), II (green), III (blue) and IV (purple). **e** Key inputs onto the LHb that contribute to the encoding of unpredicted and predicted aversive and appetitive stimulus.

Several brain structures participate in the expression of innate and learned behaviours and among those is the epithalamic lateral habenula (LHb, Fig. 1a). The LHb is an ancient brain structure that is present in virtually all vertebrate species [1] and regulates brain circuits critical for many behaviours related to motivation and decision making [2, 3]. LHb neurons are almost entirely composed of SLC17A6 (VGlut2)-expressing glutamatergic neurons, which receive synaptic inputs from a wide range of subcortical structures controlling motivation, emotion, and arousal among others [2]. The LHb is among the few brain structures that control the activity of both dopamine and serotonin neurons, which also encode positive and negative events during both innate and learned behaviours [4–6]. Therefore, the LHb locates in a strategic position in the brain, and possesses an appropriate connectivity to encode as well as predict upcoming valued external stimuli.

LHb function contributes to motor outputs, sleep and circadian rhythms and—importantly for the topic of this review—to reward and aversion encoding [2]. Indeed, LHb neurons encode rewards and punishments as well as their predictors in an opposite manner compared to dopaminergic neurons. LHb neurons are excited by reward omission or punishment and inhibited by unexpected reward or their predictors [7–9]. Notably, such an encoding is conserved across species as it is similarly present in humans, fish and rodents [2, 9–13]. If LHb function is fundamental for processing positive or negative stimuli, the prediction is that its manipulation should shape behaviours related to reward and aversion encoding. In line with this, lesioning the habenula in mice impairs various aspect of reward encoding. Among others, habenula lesion impairs downstream control of the reward system such that dopamine neurons fail to encode reward omission [14]. In addition, similar disruption of LHb function renders rats unable to display preference towards higher versus lower reward probabilities [15]. Optogenetic manipulation leading to the inhibition of LHb neuronal activity generates reward related behaviours [16, 17]. Complementary to these findings, the optogenetic activation of LHb through the heightening of glutamatergic signalling onto LHb neurons leads instead to real time place aversion [11, 18–22].

Altogether, these observations strongly support that LHb neuronal activity, as well as the incoming signalling to this structure, interfaces reward and aversion leading to the generation of adaptive behaviours. Yet, these experiments are anchored on the artificial manipulation of neuronal circuits in behavioural settings that bear little resemblance to physiological conditions. Within the next section we will discuss how innate and learned behavioural responses require similar LHb neuronal circuits, highlighting the contribution of LHb to fundamental behavioural modules.

## Lateral habenula contribution to innate behaviours

Innate behaviours emerge from the recruitment of specific programs that are genetically encoded in virtually all animal species. Innate behaviours are generally defined as inborn actions that occur in response to unconditioned stimuli and are ultimately instrumental for animal’s survival [23–27]. These behaviours can include reflex—an unconscious action that does not require the brain—and instinct, which instead requires the central nervous system. Thus, while reflex behaviours are not flexible, instinctive behaviours are the result of cognitive processes allowing the selection of an optimal action [25, 28]. In this review, we will refer to innate behaviours as those instinctive behavioural actions requiring central processing.

The selection of a reliable action is crucial for the survival of an individual and their genetic inheritance. For instance, during acute stressful events, innate defensive actions are generated in response to an external threat/aversive stimulus in order to find a solution to the dangerous situation [23, 25, 26]. The neuronal substrates underlying these behavioural programs include the amygdala, the superior colliculus, the periaqueductal grey, the hypothalamus, and the midbrain among others [29–31]. In addition, interaction between conspecifics (either pleasant or aversive) as well as parental behaviours are also innate and are integral to inheritance and newborn survival [24, 27].

Below we will discuss experimental evidence indicating how the LHb, by processing unpredicted external aversive or rewarding signals, may participate in behavioural actions stemming from threats, as well as behaviours related to social interactions.

### LHb contribution to shock-driven behavioural responses

The LHb responds to aversive external threats with a phasic increase in neuronal activity in both awake and anesthetized animals [7, 11]. This occurs independently of the nature of the negative stimulus. Indeed, using in vivo imaging and electrophysiology, foot shock, air puff and radiant heat promote excitation of the LHb [11, 32–35]. Single neuron recordings indicate however that only a subset of neurons increase in its activity after a noxious stimulus. For example, foot shock and radiant heat only excite one-third and one-half of LHb neurons, respectively [36, 37]. In addition, a minority of neurons show a phasic decrease in firing rate. This is important to note, as it may indicate that distinct subpopulations of neurons in the LHb differentially encode the same stimulus [38].

These experiments provide recordings of neuronal activity in the LHb while monitoring the behaviour of the animal. Unpredicted foot-shock exposure produces LHb excitation and concomitant escape behaviours in a context that allows the mice to shuttle to different “safe” compartments [11]. From a circuit and mechanistic standpoint this excitation may emerge through different signalling pathways converging onto LHb neurons. Experiments based on pharmacological approaches reveal that the punishment-driven excitation requires glutamate receptors expressed in LHb neurons [11]. Notably, a major source of glutamate serving for the foot-shock-driven excitation of LHb is, among others, the lateral hypothalamus [11, 18]. Reducing the efficacy of hypothalamic inputs onto the LHb through chemogenetic and optogenetic inhibition indicate that this projection is a requirement for foot-shock driven excitation of LHb neurons, and ultimately escape behaviours [11, 18, 35]. Importantly, the inhibition of such projection does not impair baseline locomotion, supporting a scenario in which LHb regulates the motivation to escape in response to a foot-shock [11]. A similar escape behaviour is observed also in zebrafish when submitted to an electrical shock [10]. Manipulating the dorsal territory of the habenula in fish, similarly to rodents, impairs the behaviour, demonstrating that this is a conserved circuit for aversion. Thus, noxious stimuli generate excitation of LHb neurons, which becomes an instrumental signal to promote a behavioural escape action across species.

### Contribution of LHb for defensive strategies

The studies discussed so far employed noxious foot-shocks to model threats and aversive experience. Yet, in a natural environment such conditions are poorly represented. When animals face a predator or an environmental threat, they engage instinctive defensive behaviours described as the “acute-stress response”. This stems from the synergistic contribution of sensory, emotion and motor circuits. The primary reaction of an animal in response to a threat, is to inhibit its movement [26]. This short period of immobility is relevant to collect information of the nature of the threat in order to prepare appropriate defensive strategies [25, 26]. These defensive responses include a palette of specific reactions including escape, fighting, freezing and thanatosis (“playing dead”). Studies in laboratory animals allow modelling of these behaviours by studying responses to a looming stimulus mimicking predator attack or using odor cues reminiscent of predator presence [39, 40]. These threats lead to discrete behavioural reactions including immobility, escape and freezing which can be measured and quantified.

Although structures including the amygdala, as well as the periacqueductal grey (PAG) and superior colliculus are vital for defensive behaviours, recent evidence indicate that the LHb also contributes to threat driven behavioural responses [11, 29, 31, 38]. Exposing mice to a predator or predator odor drives the expression of the immediate early gene c-Fos in the LHb [41, 42]. Notably, optogenetic activation of LHb glutamatergic terminals impinging onto the laterodorsal tegmentum (LDT) also promotes freezing behaviours [41]. The contribution of the LHb in innate fear expression and freezing behaviours remains however debated, as LHb lesions do not impair this behavioural component [43]. This provides a first evidence that the LHb can shape, at least partly, defensive strategy.

Aversive auditory and visual stimuli help to model external threats and indeed are able to produce immobility or escape behaviours—two opposite defensive strategies. Experiments employing the visual looming stimulus to mimic a predator attack show that mice remain immobile or rapidly escape to hide under an experimental shelter [38]. The use of calcium imaging with single cell resolution in behaving mice demonstrates that independently active LHb neuronal clusters exist in the LHb and participate during specific time epochs of defensive behaviours. Furthermore, the decoding analysis of this neuronal activity unraveled that certain clusters were predictive for the upcoming selection of the defensive action while others instead represented the selected action. Therefore, the LHb contains heterogeneous neuronal populations that may organize strategy switching during threat-driven defensive behaviours [38]. In addition to its role in the reaction to visual threats, the LHb can also encode auditory stimuli to guide escape behaviour. Mice actively avoid a compartment associated with white noise in a two-chamber open-field [44]. Interestingly, white noise excites both glutamatergic neurons in the medial septum and in the LHb. Such an activation of LHb neurons disappears after muscimol-mediated inactivation of the medial septum. In contrast, the optogenetic activation of septal glutamatergic neurons projecting to LHb induces a real-time place aversion [44].

Thus, the LHb computes aversive auditory and visual stimuli and participates in the shaping of defensive strategies most appropriate for the survival of the animal (Fig. 1b). It remains yet unknown the complete circuit connectivity by which the sensory stimulus reaches the habenular complex. The PAG and superior colliculus (SC) represent threat-detection modules integrating sensory and neuromodulatory signals enabling escape behaviours [25]. Similarly, the amygdala also functions as a threat-detection area and seems to gate the hierarchy between innate and learned aversive behaviours [45]. Future studies are then required to understand the sensorial component controlling LHb function as well as whether the habenular circuits crosstalk to other defensive neuronal hubs (PAG/SC or amygdala), or whether each network encodes a facet of these complex behaviours. Multisite recordings of calcium dynamics combined with circuit manipulation represent an opportunity to test whether the LHb neuronal activity occurs concomitantly or is dependent from other structures. In addition, modulation of the projections from the LHb innervating the raphe or the midbrain may provide new insights on the complex circuit mechanisms (input-LHb-output) implicating LHb in mediating defensive strategies.

### LHb control of social interaction

Another innate behavioural sphere to which the LHb can contribute concerns social behaviours. Social interactions are internally rewarding and essential for survival and mental health. Different studies demonstrate that LHb modulates conspecific interactions. Chemogenetic activation of LHb neurons reduces social interaction, and similarly optogenetic activation of PFC terminals in the LHb decreases social preference [46]. Moreover, chemogenetic inhibition of raphe-projecting LHb neurons decreases social interaction [47]. These are experiments depicting a contribution of the LHb in social behaviours, yet an understanding of the circuit mechanisms behind these findings remains incomplete. LHb neurons seem not to be active when littermates interact, and it remains unknown if the LHb participates in social interaction with a novel individual [13]. Employing strategies capable of labelling defined LHb neuronal populations either based on their genetic profile or connectivity may represent a way to isolate LHb neuronal modules that could participate in specific facets of social interactions. The LHb directly controls monoaminergic nuclei including the dopaminergic ventral tegmental area (VTA), which is crucial for social interaction [48] (Fig. 1a, b). Thus, focusing on individual LHb output circuits, likely those projecting to the midbrain, may provide a clearer contribution of this structure to social behaviours.

### LHb in aggressive behaviours

Social interactions may result, in specific cases, in aggressive behaviours. Social aggression is evolutionarily conserved and controls social hierarchies to preserve mating rights, or access to valuable resources. Thus, in several instances aggression is an innate component of social behaviours important for survival. LHb neurons are instrumental for social aggression [49–51]. Optogenetic manipulation of synaptic inputs emerging from the basal forebrain modulate aggression-based conditioned place preference [50]. Indeed excitation of GABAergic basal forebrain terminals in behaving mice concomitantly decreases LHb neuronal firing and promotes preference for an aggression-related compartment [50]. Thus, these data support a participation of LHb in encoding the value of aggression. This is an evolutionarily conserved neuronal circuit as optogenetic activation of the zebrafish LHb homologue increases the probability of losing a fight [52]. Mechanistically, the contribution of LHb in social aggression requires hypothalamic orexinergic control of Glutamate Decarboxylase 2 (GAD2)-expressing LHb neurons in mice. Optogenetic manipulation of GAD2 LHb neurons and of orexin inputs from the Lateral Hypothalamus (LH) to the LHb guide aggressive behaviours through Orexin-2 receptors [49]. In a different study, exposure of C57BL6 mice to aggressive CD1 mice resulted in LHb excitation [51]. If we consider that aggression is aversive for the harassed counterpart and rewarding instead for the aggressor, these results are in line with the general framework where the LHb is activated by an aversive stimulus and inhibited by appetitive stimulus [7, 8] (Fig. 1b). Does the response of LHb in the context of aggression represent only the encoding of rewarding and aversive stimuli in aggressor and non-aggressor respectively? How much of these responses contain an emotional component? Together with a limited understanding of whether the LHb participates in defensive strategies when facing aggression, these questions need further evaluation to expand our understanding of how LHb governs social interactions.

### LHb for parental and sexual behaviour

Both parental and sexual behaviours are considered a branch of innate social behaviour which are highly influenced by hormonal fluctuations. Parental behaviour relies on caring and nurturing the newborn and is essential for the survival of the species. Anatomical mapping of the neuronal areas contributing to such behaviour have identified the medial preoptic area (MPOA) and the bed nucleus of stria terminalis (BNST) as well as the reward and emotional systems as pillars for the expression of parental care [24]. In addition, pup presentation also leads to an increase in c-Fos positive neurons in the LHb and maternal behaviours, such as pup retrieval, nursing, and nest building are disrupted in mother and virgin female rodents that have a bilateral lesion of the LHb [53–55]. This role of LHb in maternal behaviour (Fig. 1b) is likely independent from hormonal changes. Newborn care is not enhanced when LHb is chronically infused with progesterone, whereas such manipulation in the MPOA stimulates maternal behaviours [56]. In addition to parental behaviours, sexual behaviour also relies, at least partly, on LHb activity and hormonal cycle. Indeed, lesions of LHb impaired sexual behaviour, such as lordosis, in female rodents while progesterone implants in the LHb stimulate lordosis and solicitation in estradiol-primed females [57–59]. The experimental evidence around these topics remains however limited, therefore further effort is necessary to understand the role of LHb in parental care and sexual behaviour.

In conclusion, although LHb neuronal responses to a variety of aversive and appetitive stimuli are binary (excitation or inhibition respectively), the LHb contributes to the expression of a diverse spectrum of innate behaviours spanning from escape to social aggression.

The next section will instead discuss LHb participation in learning processes enabling anticipation of a positive or a negative event.

## Learning behaviours guided by lateral habenula

Aside from innate behaviours, animals complement their behavioural repertoire through experience. Indeed, behaviourally adapting to the environment by learning from experience is a fundamental ability for optimizing future actions. Associative learning is one of the most basic yet crucial forms of learning in all types of adaptive behaviours. It consists of associating a neutral environmental stimulus with a rewarding or aversive experience, a process that confers on individuals the ability to predict outcomes and shape their actions accordingly. Rewards and punishments dictate this form of learning and ultimately lead to approach or avoidance behaviours respectively [30, 31, 60]. The LHb is instrumental for processing rewarding and aversive stimuli and it is central in directing behavioural adaptations that rely on reward- or aversive-based learning.

This part of the review will describe how the LHb participates in behaviours stemming from learning processes. For clarity and simplicity, we will discuss first LHb recruitment in reward-based and finally in aversive-based learned behaviours. For each section, the focus will be placed on how LHb activity represents external events and after, how it dictates behavioural adaptations.

### LHb neuronal dynamics during reward-based learning

Electrophysiological recordings in monkeys undergoing a reward-based saccade or a classic conditioning task showed that the majority of LHb neurons reduce their neuronal activity upon unexpected rewards (a drop of juice) and by reward-predicting cues (visual stimuli). The neuronal inhibition is proportional to the probability that the cue has in predicting the upcoming rewards [7, 8]. In contrast, most LHb neurons increase in their neuronal activity after the presentation of stimuli predicting reward absence or by reward omissions [7, 8]. Thus, the LHb can encode negative reward prediction error (RPE). Other studies supported these findings and confirmed that the LHb is inhibited by reward predictive cues and activated by reward omission-like events in rodents and humans [9, 12, 61]. However, this general picture becomes confusing when considering LHb responses to fully predicted rewards. Indeed, differently to what described in monkeys, the LHb, in mice, does not display a perfect negative RPE as neurons remain inhibited by predicted rewards [13, 61]. Moreover, in approximately half of all LHb cells, an excitation builds up immediately after reward consumption in mice [13]. This may arise as a rebound excitation promoted by reward-driven inhibition, but its biological significance remains unknown. In summary, these data support that LHb neuronal activity contributes to associative processes linking an initially neutral stimuli with rewards.

LHb neuronal activity not only supports learning processes but may also encode animals’ performance in reward-based behavioural tasks. Indeed, saccade latency, the number of anticipatory lickings, locomotor activity and the number of errors in a T-maze task correlates with LHb neuronal activity recorded with optical and electrophysiological techniques [7–9, 13]. This is further supported by optogenetic experiments designed to perturb LHb function by inhibiting or exciting axonal projections from different inputs. The optogenetic inhibition of terminals from the entopeduncular nucleus (EPN) to the LHb biases the mouse to nose poke in the light-paired reward port, an effect that was opposite with optogenetic activation [62]. In addition, while optogenetic activation of hypothalamic terminals is less reinforcing, the same intervention onto midbrain terminals reinforces actions [16, 21]. Although these evidence indicate that modulating LHb function biases reinforcement-based behaviours, the paradigms employed are somehow different challenging the establishment of a consensus for the role of LHb in such a behavioural outcomes [16, 18, 62, 63]. Yet, these optogenetic experiments shed some light on the input-output LHb relationships that are participating in learned appetitive behaviours (Fig. 1c, d). In addition, electrophysiological and two-photon recordings demonstrate functional diversity between LHb neuronal populations responding to reward-related signals [7, 8, 13, 61]. Although, on average, LHb neurons display responses to rewards, reward predictive cues and omissions, only a fraction of the whole LHb population (~38%) dictate this pattern [8]. Moreover, about 28% of the neurons recorded showed significant conditioned stimulus and unconditioned stimulus responses in the same directions, whereas almost ~10% of the neurons showed significant conditioned stimulus and unconditioned stimulus responses in opposite directions [8] (Fig. 1c). Therefore, merging the circuit connectivity knowledge with these functional experiments indicates that different input-output modules in LHb may independently contribute to distinct phases of reward learning processes.

### Circuit connectivity for LHb implication in reward learning

The initial work carried out by Matsumoto & Hikosaka [7] suggests that LHb activity has repercussions onto VTA dopamine neurons. LHb neuronal dynamics are diametrically opposed to those of VTA dopamine neurons recorded within the same animal after reward experience. In addition, the responses of LHb and dopamine neurons change similarly after the reversal of position-reward contingency, but in opposite directions. Response latency analysis shows that VTA dopamine responses follow those of LHb neurons, a finding complemented by data showing that electrical stimulation of the LHb drives inhibition of dopamine neurons. These observations laid the foundation for the hypothesis that the LHb drives the expression of negative RPE in VTA dopamine neurons through inhibitory neurons located within the rostromedial tegmental nucleus (RMTg) [2]. A series of experiments using silencing or lesioning of the LHb indicate that such a intervention impairs mice and rats in their ability to efficiently discriminate between high probability-delivered rewards versus low probability of reward presentation [14, 64]. This effect required the LHb-RMTg-VTA circuitry as these results are recapitulated by inactivation of the RMTg but not dorsal raphe [64]. Moreover, after LHb lesion, the VTA dopamine inhibitory responses caused by reward omission are greatly diminished and dopamine neurons’ ability to signal graded levels of positive RPEs become inconsistent [14].

Taken together, this experimental evidence supports a scenario in which the LHb-RMTg-VTA dopamine output circuit contributes to reward-based learned behaviours. On the other hand, which synaptic inputs impinging onto the LHb participate in these behaviours remains less clear. Optogenetic experiments highlight different structures projecting to the LHb such as VP, EPN and VTA, among others (Fig. 1d). Some plausible inhibitory inputs such as VTA and lateral preoptic area (LPO) might contribute to LHb encoding of classical appetitive conditioning given that their manipulation can modulate reward-related behaviours. VTA VGlut2/VGAT neurons projecting to LHb respond to both expected and unexpected rewards but not to reward predictive cues or reward omissions, suggesting that they do not contribute to the learned aspect of this behaviour [65]. Moreover, LPO VGAT neurons projecting to LHb do not respond to either reward consumption or reward predictive cues [22]. Two other possible inputs are the EPN and the VP neurons. Indeed, different reports show excitation of EPN in rodents or GPi in primates upon reward cues and rewards [19, 62, 66]. In addition, EPN inactivation in rats completely abolishes LHb responses to reward predictive cues [67]. It is important to note however that this structure co-releases both glutamate and gamma-Aminobutyric acid (GABA) with a net excitatory signalling [19]. Thus, LHb inhibitory responses could be explained by a reduced excitatory drive rather than an increase in phasic release of GABA [62, 66]. Lastly, VP GABAergic neurons also represent a good candidate to drive inhibitory responses in LHb neurons for reward encoding and appetitive learning. This neuronal population projecting to the LHb responds with an excitation to cues and rewards, and its optogenetic modulation bi-directionally modulates animals’ movements towards rewards [63]. These findings help to build an anatomical framework of structures contributing to different aspects of reward-related learning and highlight how neuronal activity in the LHb is embedded in this reward module. The missing aspect of this model, however, remains the causality between LHb neuronal activity and reward related behaviours. There are, at the moment, no experimental evidence demonstrating which phase of reward learning engages LHb neurons in order to be instrumental for the behavioural outcome. Combining imaging approaches, electrophysiology and manipulation strategies may enable us to understand when LHb is relevant in reward behaviours and through which circuits and mechanisms.

### Contribution of the LHb in aversive learning

LHb population activity in a pavlovian conditioning task in monkeys suggests that this structure encodes aversive stimuli and their predictors [8]. Seminal studies showed that when monkeys are presented with different cues predicting an aversive stimulus (airpuff) with different probabilities (100, 50 and 0%), the magnitude of neuronal activity increases after the appearance of the 100% and 50% airpuff conditioned stimuli and decreases after the appearance of the 0% airpuff conditioned stimulus. Moreover, the magnitude of the response to the aversive stimulus is inversely proportional to its probability to be delivered. Indeed, the change in LHb neuron activity is greatest when the unconditioned aversive stimulus is totally unexpected and the smallest when it is fully predicted [8]. The emergence of excitatory responses to cues predicting aversive stimuli occurs independently of the nature of the aversive stimulus as it was recapitulated with quinine, foot-shock and social aggression [10, 13, 35, 67]. Notably, LHb neuronal responses to the unconditioned stimulus in mice remain stable even after the emergence of conditioned stimulus-driven excitation [13, 35]. This may indicate that the LHb does not encode a “classic” prediction error but rather it represents an absolute value of the aversive stimuli and their predictive cues.

Similarly to reward-related tasks, LHb neuronal activity reflects animals’ behavioural performance in aversive-based learning. Indeed, different behavioural readouts such anticipatory blinking, locomotor activity and avoidance rate, confirm how variations in LHb activity directly correspond with changes in behavioural outcome [8, 13, 35].

Altogether, these studies highlight the fundamental role the LHb has in mediating the learning processes related to aversive experience.

Accordingly, LHb neuronal activity contributes to aversive learning processes such as avoidance. LHb inactivation via muscimol infusion disrupts the stability of avoidance memories [68]. In addition LHb pharmacological inactivation impairs active defensive responses biasing behaviours toward reward seeking, when threats and safe memories are in conflict [69]. Furthermore, hypothalamic to LHb terminal activity during conditioned stimulus presentation, and AMPA receptor potentiation at LH receiving synapses in LHb, is crucial for mice to learn to avoid an imminent punishment [35, 70, 71]. Similarly, in zebrafish, inhibiting ventral habenula terminals in median raphe impairs avoidance learning, but leaves intact panic behaviour induced by classical fear conditioning [10]. These data, however, contrast with the observation that LHb lesions in rats leads to better avoidance performance [71].

While the LHb contribution to encoding of unpredicted and predicted threats is established by considerable evidence, its participation in classical threat-driven fear responses (classical fear conditioning) remains debated. For instance, Durieux et al. [72] show that LHb chemogenetic inhibition during the conditioning phase does not affect rats’ freezing behaviour to cue presentation. On the other hand, this intervention reduced freezing during the re-exposure of rats to the context, yet increased freezing to the cue, previously associated with footshock—demonstrating contextual memory deficits and enhanced fear to the tone, respectively, following LHb inhibition [72].

### Circuit connectivity underlying learning to aversive stimuli

The working model arising from the data described above supports the idea that connectivity and input-output relationships explain the LHb contribution to aversive-based learned behaviours. The LHb receives innervation from a variety of brain structures that create a neuronal network for aversive-based learned behaviours. Pharmacological and optogenetic manipulation of LHb inputs from the EPN support the contribution of this pathway for negative valence encoding [21]. Similarly, the VP and LH projections to the LHb also control encoding of reward and aversion [18, 62]. The hypothalamic relevance for control of LHb neuronal activity in learned behaviours is further supported by a series of experiments demonstrating that this pathway is instrumental for avoidance learning [35]. Indeed, experiments employing optogenetic silencing of this pathway together with manipulation of excitatory transmission at hypothalamic synapses onto the LHb have helped to elucidate circuit and mechanistic insights for the instrumental role of this pathway in learning to anticipate an aversive event [35].

Other inputs onto LHb may also contribute to aversive conditioning. For instance, optogenetic stimulation of afferent terminals of the LPO and the VTA onto the LHb elicits conditioned place aversion in mice [28, 73]. It remains unknown, however, whether these inputs participate in learning about aversive stimuli, or whether the optogenetic stimulation biases the function of the LHb.

Less explored are the output regions of LHb neurons participating in aversive-learned behaviours. Optogenetic activation of LHb terminals in VTA is sufficient to elicit conditioned place aversion in mice by downstream activation of dopaminergic neurons projecting to the medial prefrontal cortex [74]. In addition, optogenetic activation of LHb terminals in the RMTg also elicits conditioned place aversion and promotes negative reinforcement [20].

Altogether, these studies consistently support that the LHb represents a fundamental brain structure for guiding learning of approach/avoidance behaviours. However, an understanding of how different inputs onto this structure work in concert to ultimately instruct LHb activity during the learning process remains elusive. In addition, whether the role of the LHb in guiding reward/aversive-based learning also has a basis in specific genetically defined sub-populations remains unclear. It is likely that incorporating both circuit and genetic determinants can help to ultimately resolve the LHb subsystems dedicated to independent aspects of learned behaviours (Fig. 1c, d). Although, recent studies have started unraveling some genetic features related to the output circuitry of the LHb [75, 76], further work is needed to bring this understanding to the next level and describe the functional properties of these genetically defined populations.

## Conclusions and perspectives

Animals need to generate appropriate actions in order to maximize reward and minimize punishment. The evidence discussed in this review supports a framework in which the LHb shapes behavioural responses that are both innate and learned after experience.

While a large portion of the literature describes a variety of mechanisms, and circuits associated to how the LHb shapes aversive behaviours, much less is known about the rules by which LHb supports reward processing. Some indications suggest that the LHb responds to rewarding stimuli and participate in reward seeking [14, 15]. However, information on the activity dynamics in the LHb underlying reward behaviours, or the potential synaptic adaptations supporting these process remains unknown. Future work should aim to understand whether neuronal populations encoding threats and reward overlap or are functionally independent. This is relevant as (i) responses to unpredicted or conditioned aversive stimuli are diverse across a large neuronal population (not simply inhibited by rewards or excited by aversive stimuli) [67], (ii) Threats can differentially engage LHb neuronal activity depending on the ultimate behavioural action [38] and (iii) Foot-shocks can excite a portion of LHb neurons whilst inhibiting another [20, 36]. The integration of functional approaches including endoscope or 2-photon calcium imaging together with behavioural tasks modelling seeking or avoidance would set the perfect conditions to tackle these questions.

This functional diversity further stresses the need for a deeper understanding of the genetic/molecular diversity present in the LHb. Independent genetic clusters of LHb neurons exist and Act-sequencing procedures show that subsets of identified neuronal clusters display elevated Immediate Early Gene expression following foot-shock exposure [75, 76]. Therefore, several genetically identified cell types in the LHb merit attention in future studies to understand whether stimuli of different nature engage similar or independent cell populations in the LHb.

Currently, we are likely only exploring the tip of the iceberg in terms of understanding the circuitry and neuronal cell types of the LHb. To provide more details and understand the complexities of LHb function in this area, it is mandatory to combine longitudinal recordings or imaging from genetically defined LHb neuronal populations during unpredicted stimuli presentation, as well as classical and operant conditioning procedures. Furthermore, expanding knowledge of the behavioural repertoires in which the LHb may participate will also give an indication under which conditions these cells are recruited. These studies will certainly reveal distinct response profiles within these populations that may or may not segregate anatomically. It will then be essential to combine the functional approaches with input and output connectivity of these subpopulations to effectively establish their function in relationship to the larger circuits of the brain implicated in innate and learned behaviours.
