# Correction: Reward and aversion encoding in the lateral habenula for innate and learned behaviours

**DOI:** 10.1038/s41398-022-01857-6

**Published:** 2022-02-28

**Authors:** Sarah Mondoloni, Manuel Mameli, Mauro Congiu

**Affiliations:** 1 The Department of Fundamental Neuroscience, The University of Lausanne, 1005 Lausanne, Switzerland; 2 Inserm, UMR-S 839, 75005 Paris, France

**Keywords:** Learning and memory, Molecular neuroscience

Correction to: *Translational Psychiatry* 10.1038/s41398-021-01774-0, published online 10 January 2022

The original version of this article unfortunately contained a mistake. It appears the authors forgot to convert a citation into the correct format and consequently omitted it from the references. The correct reference is: Velazquez-Hernandez, G, Sotres-Bayon, F. Lateral habenula mediates defensive responses only when threat and safety memories are in conflict. eNeuro. 2021;8;ENEURO.0482-20.2021. 10.1523/ENEURO.0482-20.2021. The original article has been corrected.

